# A novel clinical tool and risk stratification system for predicting the event-free survival of neuroblastoma patients: A TARGET-based study

**DOI:** 10.1097/MD.0000000000034925

**Published:** 2023-09-22

**Authors:** Mingzhen Li, Xiaoying Duan, Chunyan Li, Di You, Linlin Liu

**Affiliations:** a Department of Radiation Oncology, China-Japan Union Hospital of Jilin University, Nanguan District, Changchun, Jilin, People’s Republic of China; b Department of Acupuncture and moxibustion, Second Hospital of Jilin University, Nanguan District, Changchun, Jilin, People’s Republic of China; c Department of Endocrinology, The Affiliated Hospital of Beihua University, Chuanying District, Jilin, People’s Republic of China; d Department of Anesthesiology, China-Japan Union Hospital of Jilin University, Nanguan District, Changchun, Jilin, People’s Republic of China.

**Keywords:** event-free survival, neuroblastoma, nomogram, prognostic model, risk stratification system, TARGET

## Abstract

Neuroblastoma (NB), considered the most common non-intracranial solid tumor in children, accounts for nearly 8% of pediatric malignancies. This study aimed to develop a simple and practical nomogram to predict event-free survival (EFS) in NB patients and establish a new risk stratification system. In this study, 763 patients primarily diagnosed with NB in the Therapeutically Applicable Research to Generate Effective Treatments (TARGET) database were included and randomly assigned to a training set (70%) and a validation set (30%) in a 7:3 ratio. First, the independent prognostic factors of EFS for NB patients were identified through univariate and multivariate Cox regression analyses. Second, a nomogram was created based on these factors and was validated for calibration capability, discriminative, and clinical significance by C-curves, receiver operating characteristic (ROC) curves, and decision curve analysis. Finally, a new risk stratification system was established for NB patients based on the nomogram. The univariate Cox analysis demonstrated that NB patients with age at diagnosis >318 days, International Neuroblastoma Staging System (INSS) stage 4, DNA diploidy, MYCN amplification status, and children oncology group (COG) high-risk group had a relatively poor prognosis. However, according to the multivariate Cox regression analysis, only age, INSS stage, and DNA ploidy were independent predictive factors in NB patients regarding EFS, and a nomogram was created based on these factors. The area under the curve (AUC) values of the ROC curves for the 3-, 5-, and 10-year EFS of this nomogram were 0.681, 0.706, and 0.720, respectively. Additionally, the AUC values of individual independent prognostic factors of EFS were lower than those of the nomogram, suggesting that the developed nomogram had a higher predictive reliability for prognosis. In addition, a new risk stratification system was developed to better stratify NB patients and provide clinical practitioners with a better reference for clinical decision-making. NB patients’ EFS could be predicted more accurately and easily through the constructed nomogram and event-occurrence risk stratification system, allowing clinicians to better differentiate NB patients and establish individualized treatment plans to maximize patient benefits.

## 1. Introduction

Neuroblastomas (NB) are tumors originating from primitive neural crest cells and occurring anywhere along the sympathetic nervous system chain.^[1]^ The most common primary site of NB is the adrenal medulla (40%) and abdominal ganglia (25%), but NB can also occur in the sympathetic ganglia of the thorax (15%), pelvis (5%), and neck (3%–5%).^[2]^ The histologic types of malignant NB can be divided into ganglioneuroblastoma (GNB) and NB.^[3]^ NB is the most common extracranial solid tumor in children, accounting for approximately 8% of all of the malignancies in children, with an incidence rate of approximately 0.013%–0.014%, and causing 15% of cancer-related deaths in pediatric patients.^[4,5]^ Approximately 95% of NB and GNB occur in patients <5 years of age, and the median age of NB patients at first diagnosis is approximately 19 months.^[6]^

As a heterogeneous disease, an individual prognosis for NB varies depending upon his/her tumor stage, tumor size, age at diagnosis, and biological features.^[7]^ The clinical course of patients with NB may be characterized by spontaneous tumor regression or cured with surgery alone; it may also be characterized by inevitable death of patients despite receiving systemic treatment.^[8]^ The 2 common staging systems for NB are the International Neuroblastoma Staging System (INSS), developed in 1988, and the International Neuroblastoma Risk Group Staging System, released in 2009. The former staging criteria are mainly based on the surgical treatment of NB; therefore, the same tumor can be staged according to the extent of surgical resection.^[9]^ The INSS is widely used for the clinical staging of primary organs and metastases of NB. While INGRSS is a preoperative risk stratification system for NB patients based on age at diagnosis, histological type, tumor grade, MYCN amplification status, 11q aberration, and DNA ploidy, and NB patients are divided into 4 risk groups accordingly: high risk, intermediate risk, low risk, and very low risk.^[10]^ In addition, the children oncology group (COG) system is often used as a reference in making clinical treatment decisions. It classifies the risk of NB patients into 3 risk groups based on INSS staging, age at diagnosis, MYCN status, histological type, and DNA ploidy status.^[11]^

These staging systems are primarily used to develop treatment plans for patients and cannot be directly used to predict the expected overall survival (OS) of NB patients. The results of a prognostic study conducted by Li et al revealed that age >520 days, INSS stage 4, and DNA ploidy were independent risk factors of OS for pediatric NB patients; these results were used to construct a prognostic model for NB patients based on these 3 factors.^[12]^ In 2020, Chen et al depicted that age, tumor stage, radiotherapy, and surgery were independent prognostic risk factors of OS for adrenal NB patients.^[13]^ In 2023, Chen et al developed a nomogram based on the age, primary tumor site, tumor size, stage, and therapeutic management to predict the cancer-specific survival of NB patients.^[14]^ It is fair to say that studies about OS in NB patients are not uncommon, but fewer scholars have focused on event-free survival (EFS) in NB patients.

The Therapeutically Applicable Research to Generate Effective Treatments (TARGET) database is an open database provided by the National Cancer Institute (NCI); it contains relevant data for a wide range of pediatric tumors.^[15]^ This study aimed to extract data on NB patients with specific clinical information from this database and analyze the independent prognostic factors affecting NB patients’ EFS to assess the prognosis of NB patients more accurately. Subsequently, a nomogram was developed to predict the 3-, 5-, and 10-year EFS of NB patients, and a new risk stratification system was developed based on this nomogram.

## 2. Method

### 2.1. Database

All the patient data included in our study was obtained from the TARGET database. As TARGET is a publicly available database and the data collected do not include explicit information about individual patients, this study did not require approval from the ethics committee or informed consent from the considered patients. This study followed the STROCSS 2021 standard.^[16]^

### 2.2. Patient selection

**Figure 1.**
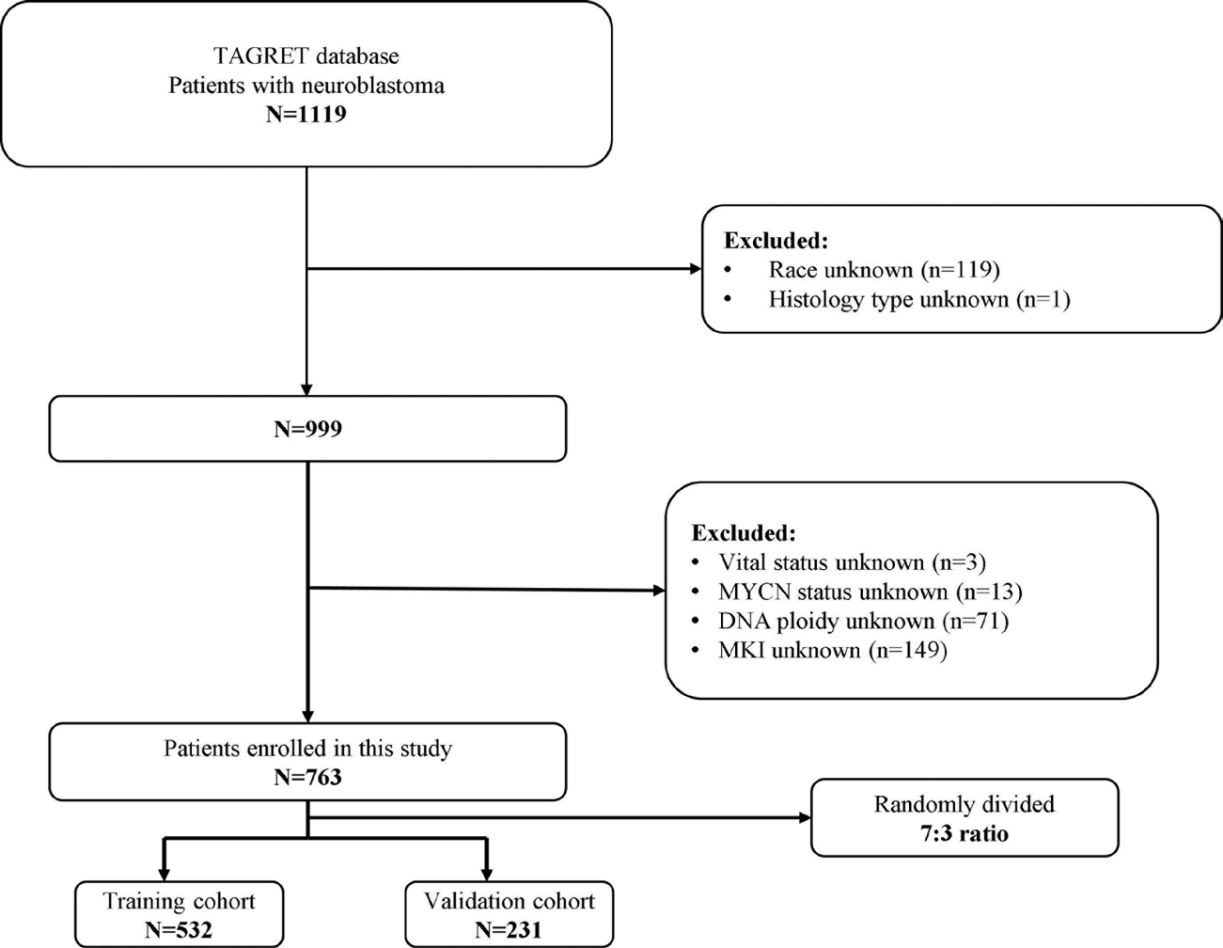
Flowchart of patient selection.

The inclusion criteria for patient selection were as follows: the patient primary tumor was NB; the histologic type was NB or ganglioneuroblastoma; and complete follow-up information was available. The exclusion criteria for patient selection were as follows: the patient survival status and EFS time were unknown; race, gender, and histological type were unknown; and MYCN status, DNA ploidy, and mitosis-karyorrhexis index (MKI) were unknown. Finally, 763 NB patients were included in the study and randomly divided in a 7:3 ratio into the training set (n = 532, 70.0%) and the validation set (n = 231, 30.0%). The training set was analyzed to obtain the independent prognostic predictors and create a predictive nomogram and a new event-occurrence risk stratification system based on the prognostic factors. The validation set was then used to verify the predictive reliability and accuracy of the nomogram and the risk stratification system (Fig. 1).

### 2.3. Variable definition

Eleven variables were selected for this study as follows: epidemiological characteristics of patients (age, gender, and race); disease characteristics (tumor histological type, MKI, INSS stage, tumor grade, tumor site, and COG risk grouping); and genetic characteristics (MYCN status and DNA ploidy). The optimal age cutoff values for NB patients were obtained from the X-tile software, and the results demonstrated that 318 and 1425 days were the best cutoff values (Supplementary Figure 1, http://links.lww.com/MD/J630). Gender was categorized as male and female. Race was divided into black, white, and other. Histological type was divided into NB and ganglioneuroblastoma. The MKI and COG risk groups were respectively classified into low, middle, and high groups. The INSS stage was divided into stage 1, 2, 3, 4, and 4s. Tumor grade was divided into differentiated and undifferentiated or poorly differentiated. Tumor site was classified as adrenal, abdominal, thoracic, and other sites. MYCN status was classified as amplified and unamplified. DNA ploidy was classified as diploid and hyperdiploid. The endpoint of this study was EFS, which was defined as the time duration from the day of formal diagnosis to the occurrence of a significant event (death, tumor recurrence, or tumor progression) in the patients.

### 2.4. Data analysis

All of the data analyses in this study were done using the SPSS (27.0) and R (4.2.1) software, and *P* < .05 was deemed statistically significant. First, specific values were individually assigned to the variables included in this study, and a table for the basic epidemiological and clinicopathological characteristics of NB patients was constructed. Second, the statistically significant variables were obtained as a result, and the univariate and multivariate Cox regression analysis and KM survival curves for each variable were plotted. Third, a nomogram was constructed based on the independent predictors acquired to predict the 3-, 5- and 10-year EFS for NB patients. In addition, calibration and receiver operating characteristic (ROC) curves were developed to verify the calibration accuracy and the discriminatory ability of the nomogram for the calculation of 3-, 5- and 10-year EFS in NB patients, respectively. Furthermore, decision curve analysis was applied to measure the value of clinical application. Additionally, the event-occurrence risk score of patients was obtained by summing the scores of each predictive factor, and the most suitable cutoff point for the risk score was determined through the X-tile (3.6.1) software (Supplementary Figure 2, http://links.lww.com/MD/J631). Finally, the risk score was used to classify the NB patients into the high-risk, middle-risk, and low-risk subgroups, and KM curves were established to demonstrate the differences in EFS among the 3 risk groups.

## 3. Result

### 3.1. Epidemiological and clinicopathological characteristics

In all, 763 NB patients from the TARGET program database were enrolled as the primary data for this study, and we randomly assigned the patients to the training set (n = 532, 70.0%) and the validation set (n = 231, 30.0%) in a 7:3 ratio by the R software. A majority of the patients with NB were white (n = 630, 82.5%), diagnosis age 318 to 1425 days (n = 418, 54.8%), and the proportion of male (n = 443, 58.1%) patients was slightly higher than that of female patients (n = 320, 41.9%). Most patients were primarily diagnosed as INSS stage 4 (n = 512, 67.1%) and were classified as COG high-risk subgroups (n = 531, 69.6%). As for the histological type, NB (n = 685, 89.8%) was more common than ganglioneuroblastoma (n = 78, 10.2%), while the tumor grade was mostly undifferentiated or poorly differentiated (n = 713, 93.4%), and there were few differences among the 3 MKI groups. Statistically, the most common primary sites of NB were adrenal (328, 43.0%), while others could occur in abdominal (n = 275, 36.0%), thoracic (n = 92, 12.1%) sympathetic ganglia, and other places (n = 68, 8.9%). Furthermore, 29.4% of the patients (n = 224) had MYCN gene amplification, and 36.4% (n = 278) had DNA diploid tumors (Table 1).

**Table 1:** Epidemiological and clinicopathological characteristics of NB patients.

Variables	Training cohort		Validation cohort		Total	
	532	70.0%	231	30.0%	763	100.00%
Age (d)						
<318	116	21.8%	58	25.1%	174	22.8%
318–1425	299	56.2%	119	51.5%	418	54.8%
>1425	117	22.0%	54	23.4%	171	22.4%
Race						
Black	63	11.8%	36	15.6%	99	13.0%
White	444	83.5%	186	80.5%	630	82.5%
Other	25	4.7%	9	3.9%	34	4.5%
Sex						
Male	310	58.3%	133	57.6%	443	58.1%
Female	222	41.7%	98	42.4%	320	41.9%
INSS stage						
Stage 1	44	8.3%	28	12.1%	72	9.4%
Stage 2	40	7.5%	16	6.9%	56	7.3%
Stage 3	54	10.2%	22	9.5%	76	10.0%
Stage 4	361	67.9%	151	65.4%	512	67.1%
Stage 4s	33	6.1%	14	6.1%	47	6.2%
Tumor grade						
Poorly differentiated	505	94.9%	208	90.0%	713	93.4%
Differentiating	27	5.1%	23	10.0%	50	6.6%
Histological Type						
Neuroblastoma	472	88.7%	213	92.2%	685	89.8%
Ganglioneuroblastoma	60	11.3%	18	7.8%	78	10.2%
Tumor site						
Adrenal gland	230	43.2%	98	42.4%	328	43.0%
Abdominal	197	37.1%	78	33.8%	275	36.0%
Thoracic	58	10.9%	34	14.7%	92	12.1%
Other	47	8.8%	21	9.1%	68	8.9%
MYCN status						
Not amplified	366	68.8%	173	74.9%	539	70.6%
Amplified	166	31.2%	58	25.1%	224	29.4%
Ploidy						
Diploid	206	38.7%	72	31.2%	278	36.4%
Hyperdiploid	326	61.3%	159	68.8%	485	63.6%
MKI						
Low	198	37.2%	103	44.6%	301	39.4%
Middle	165	31.0%	64	27.7%	229	30.1%
High	169	31.8%	64	27.7%	233	30.5%
COG risk						
Low	93	17.5%	47	20.3%	140	18.3%
Middle	60	11.3%	32	13.9%	92	12.1%
High	379	71.2%	152	65.8%	531	69.6%

COG = children oncology group, INSS = International Neuroblastoma Staging System, MKI = mitosis-karyorrhexis index, NB = neuroblastoma.

### 3.2. Independent predictive factors for EFS of NB

**Figure 2.**
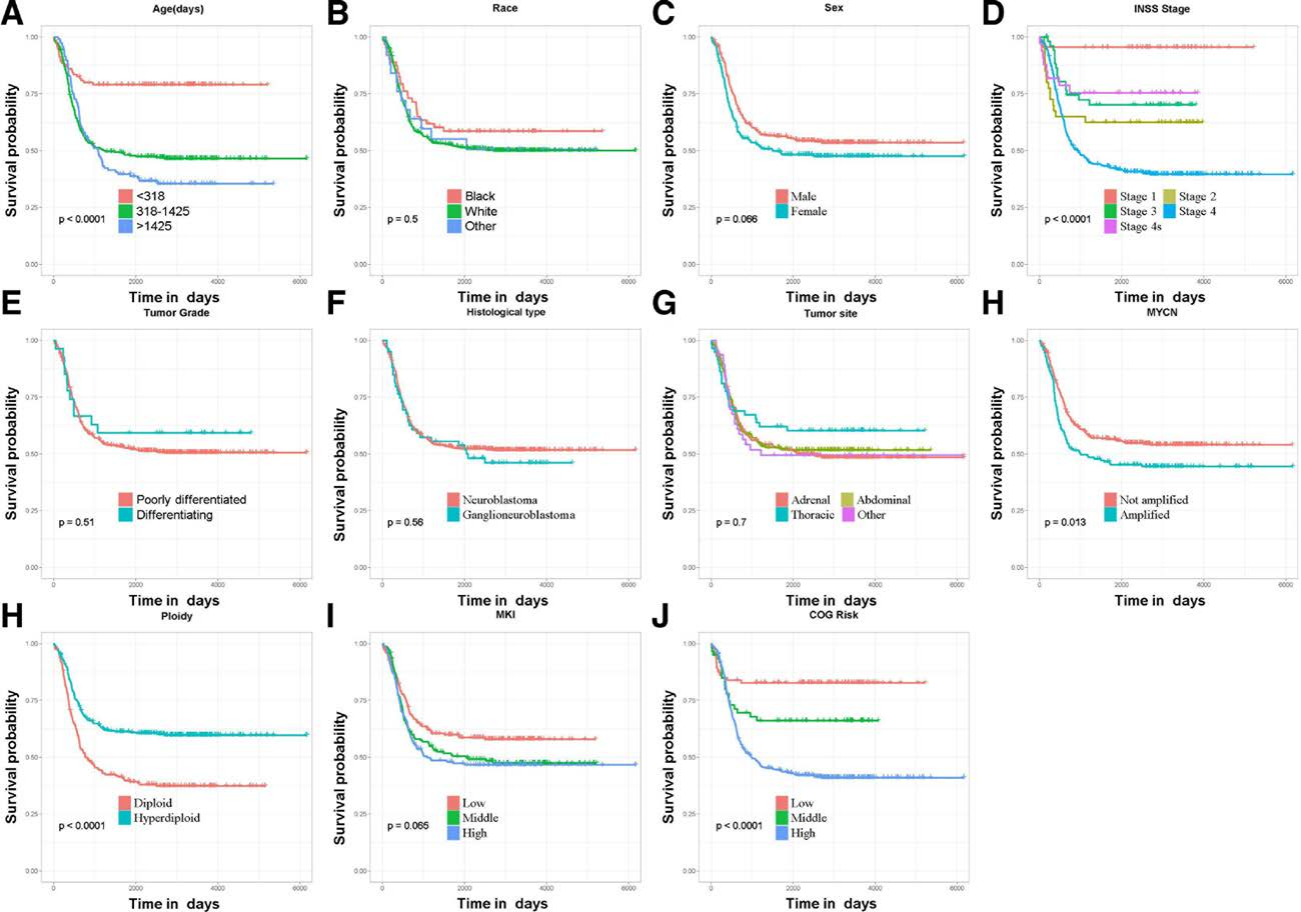
Kaplan–Meier event-free survival analysis curves of each variable. (A) Age, (B) race, (C) sex, (D) tumor INSS stage, (E) tumor grade, (F) histological type, (G) tumor site, (H) MYCN status, (I) DNA ploidy, (J) MKI, and (K) COG risk groups. COG = children oncology group, INSS = International Neuroblastoma Staging System, MKI = mitosis-karyorrhexis index.

Univariate and multivariate Cox regression analyses were performed to recognize the independent predictive factors of EFS in patients with NB. In all, 11 variables, namely age, race, gender, tumor grade, INSS stage, tumor site, histological type, COG risk group, MYCN gene status, DNA ploidy, and MKI, were analyzed in the univariate Cox analysis, and the KM curves were plotted. Age, INSS tumor stage, COG risk groups, MYCN status, and DNA ploidy were considered the EFS-related variables (*P* < .05); in the meantime, no significant differences were observed for race, gender, tumor grade, tumor site, histological type, and MKI (*P* > .05) (Fig. 2). Finally, the results of the multivariate Cox analysis demonstrated that age of diagnosis >1425 days (*P* = .044), INSS stage 4 (*P* = .005), and DNA diploid (*P* = .008) were the independent prognostic risk factors for EFS in children with NB (Table 2).

**Table 2:** Univariate and multivariate Cox regression analyses.

Variables	Univariate analysis		*P* value	Multivariate analysis		*P* value
	OR	95% CI		OR	95% CI	
Age (d)						
<318	Reference			Reference		
318–1425	3.046	1.981–4.683	≤.001	1.763	0.919–3384	.088
>1425	3.517	2.218–5.578	≤.001	2.053	1.018–4.138	.044
Race						
Black	Reference					
White	1.278	0.851–1.919	.238			
Other	1.261	0.636–2.499	.507			
Sex						
Male	Reference					
Female	1.261	0.985–1.615	.066			
INSS stage						
Stage 1	Reference			Reference		
Stage 2	11.372	2.6–49.732	.001	8.859	1.882–41.707	.006
Stage 3	7.481	1.711–32.718	.008	4.933	0.871–27.936	.071
Stage 4	18.396	4.569–74.059	≤.001	11.25	2.094–60.444	.005
Stage 4s	6.725	1.428–31.669	.016	7.203	1.462–35.497	.015
Tumor grade						
Poorly differentiated	Reference					
Differentiating	0.818	0.447–1.497	.515			
Histological Type						
Neuroblastoma	Reference					
Ganglioneuroblastoma	1.12	0.769–1.63	.556			
Tumor site						
Adrenal gland	Reference					
Abdominal	0.95	0.723–1.249	.715			
Thoracic	0.769	0.491–1.204	.251			
Other	1.023	0.654–1.602	.92			
MYCN status						
Not amplified	Reference					
Amplified	1.381	1.069–1.785	.014			
Ploidy						
Diploid	Reference			Reference		
Hyperdiploid	0.536	0.419–0.686	≤.001	0.698	0.536–0.909	.008
MKI						
Low	Reference					
Middle	1.323	0.975–1.795	.072			
High	1.402	1.037–1.894	.028			
COG risk						
Low	Reference					
Middle	2.155	1.117–4.16	.022			
High	4.071	2.448–6.768	≤.001			

CI = confidence interval, COG = children oncology group, INSS = International Neuroblastoma Staging System, MKI = mitosis-karyorrhexis index, OR = odds ratio.

### 3.3. Construction and validation of a nomogram

**Figure 3.**
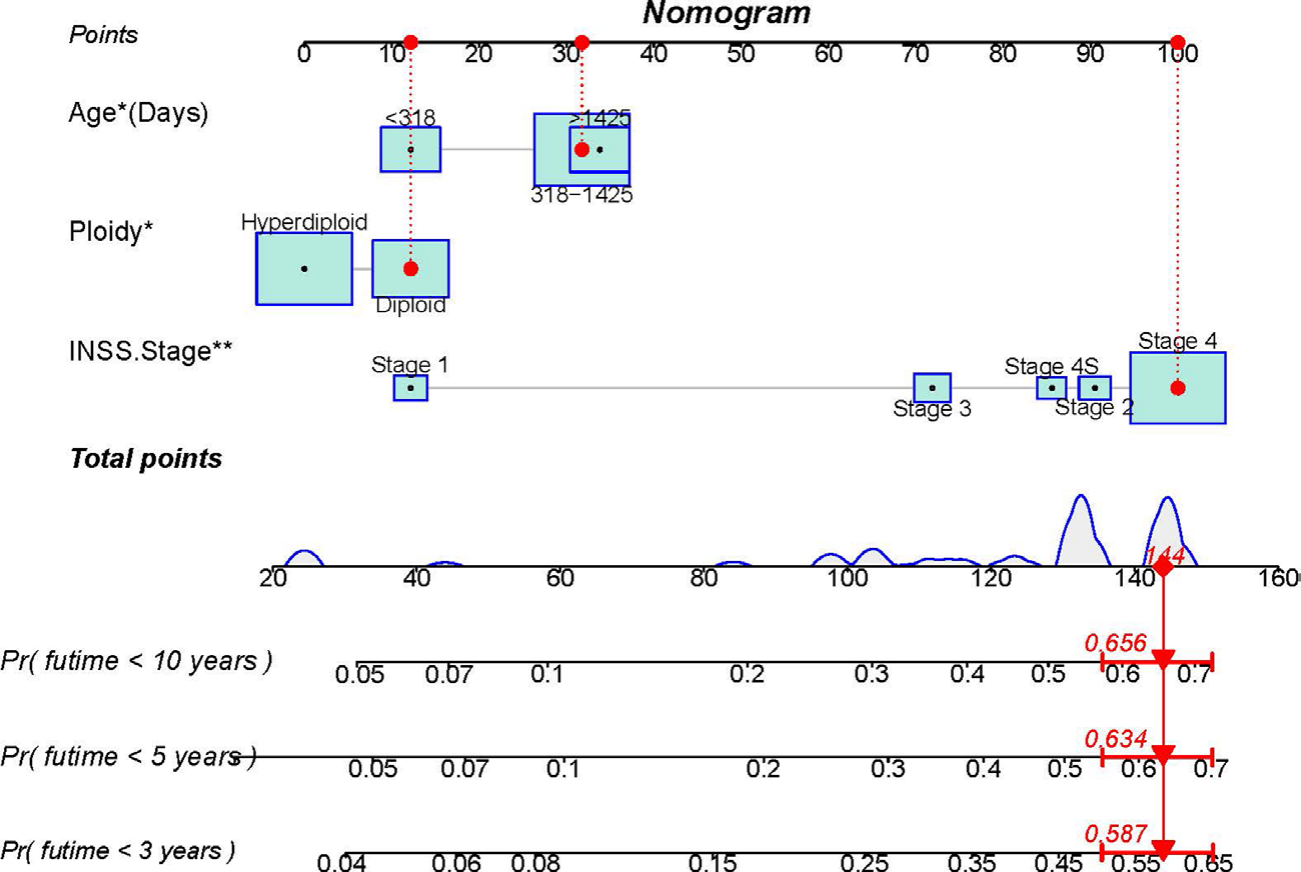
Nomogram for EFS prediction of NB patients. EFS = event-free survival, NB = neuroblastoma.

**Figure 4.**
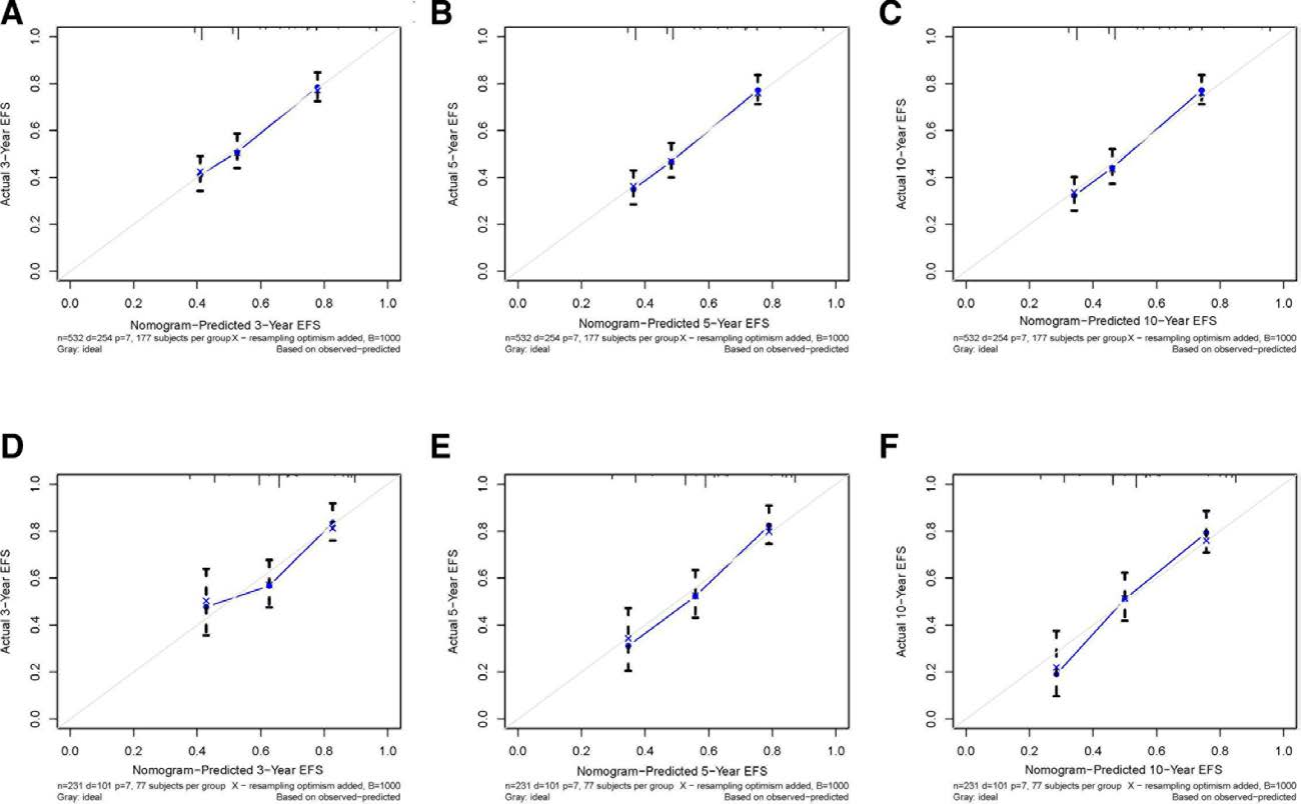
Calibration curves of the nomogram. (A–C) 3-, 5-, and 10-yr EFS in the training set. (D–F) 3-, 5-, and 10-yr EFS in the validation set. EFS = event-free survival.

**Figure 5.**
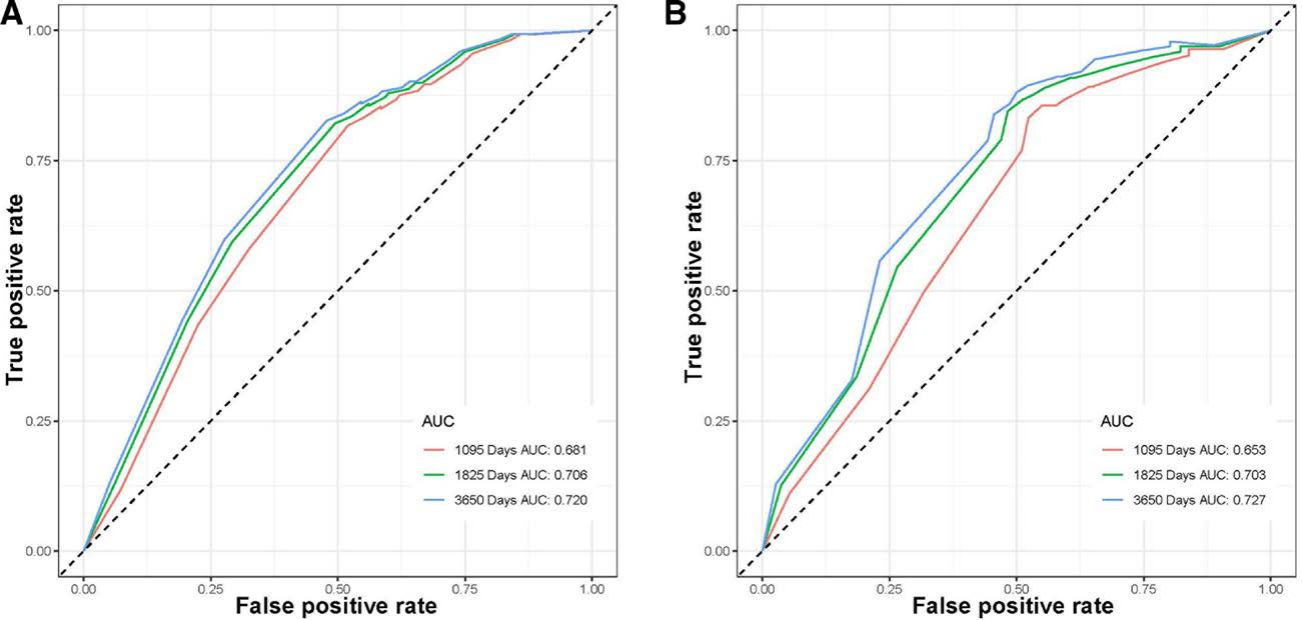
Receiver operating characteristic (ROC) curves of NB patients. (A) Training set and (B) validation set. NB = neuroblastoma, ROC = receiver operating characteristic.

**Figure 6.**
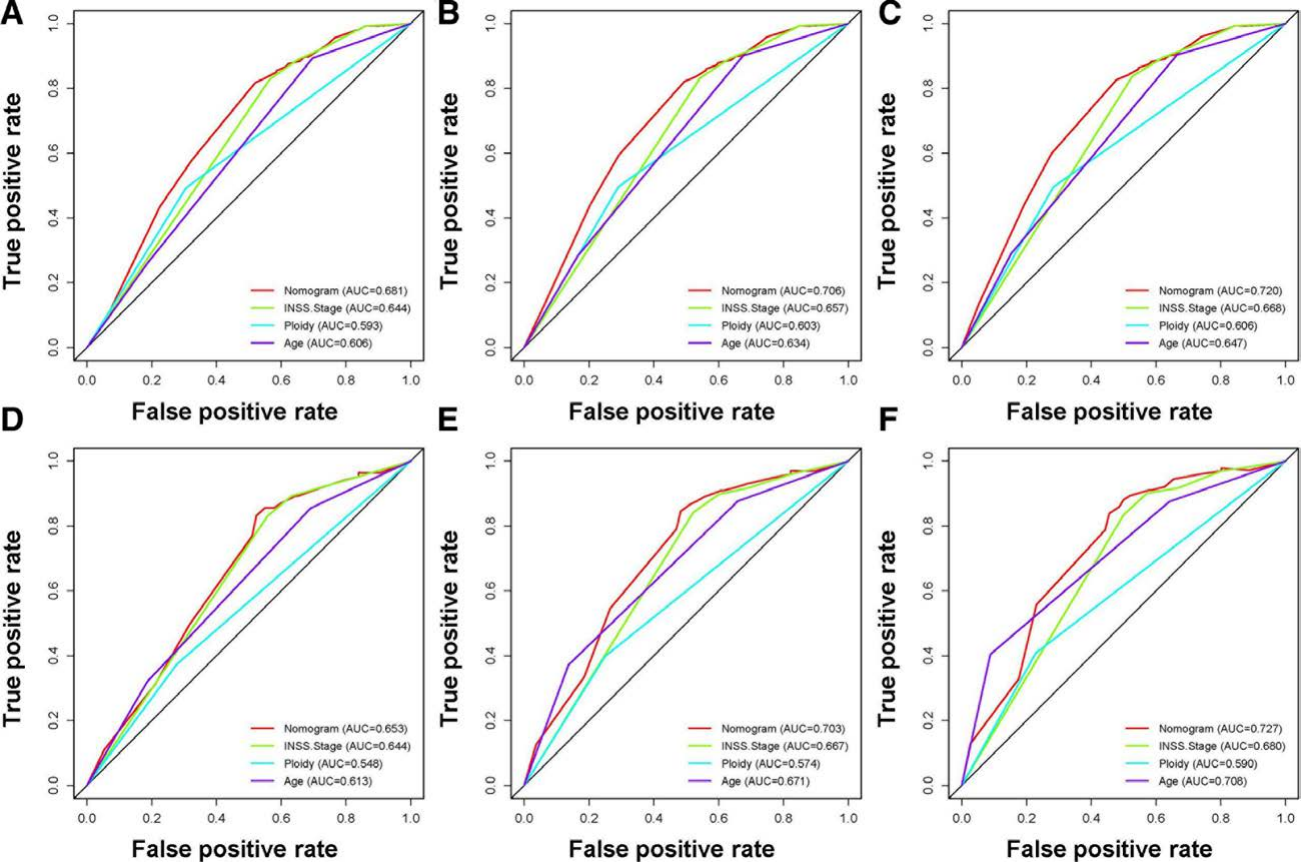
Comparison of forecast ability (AUCs) between the constructed nomogram and separate EFS-related predictors in this study. (A–C) the ROC curves of 3-, 5-, and 10-yr EFS in the training set; and (D–F) the ROC curves of 3-, 5-, and 10-yr EFS in the validation set. EFS = event-free survival, ROC = receiver operating characteristic.

**Figure 7.**
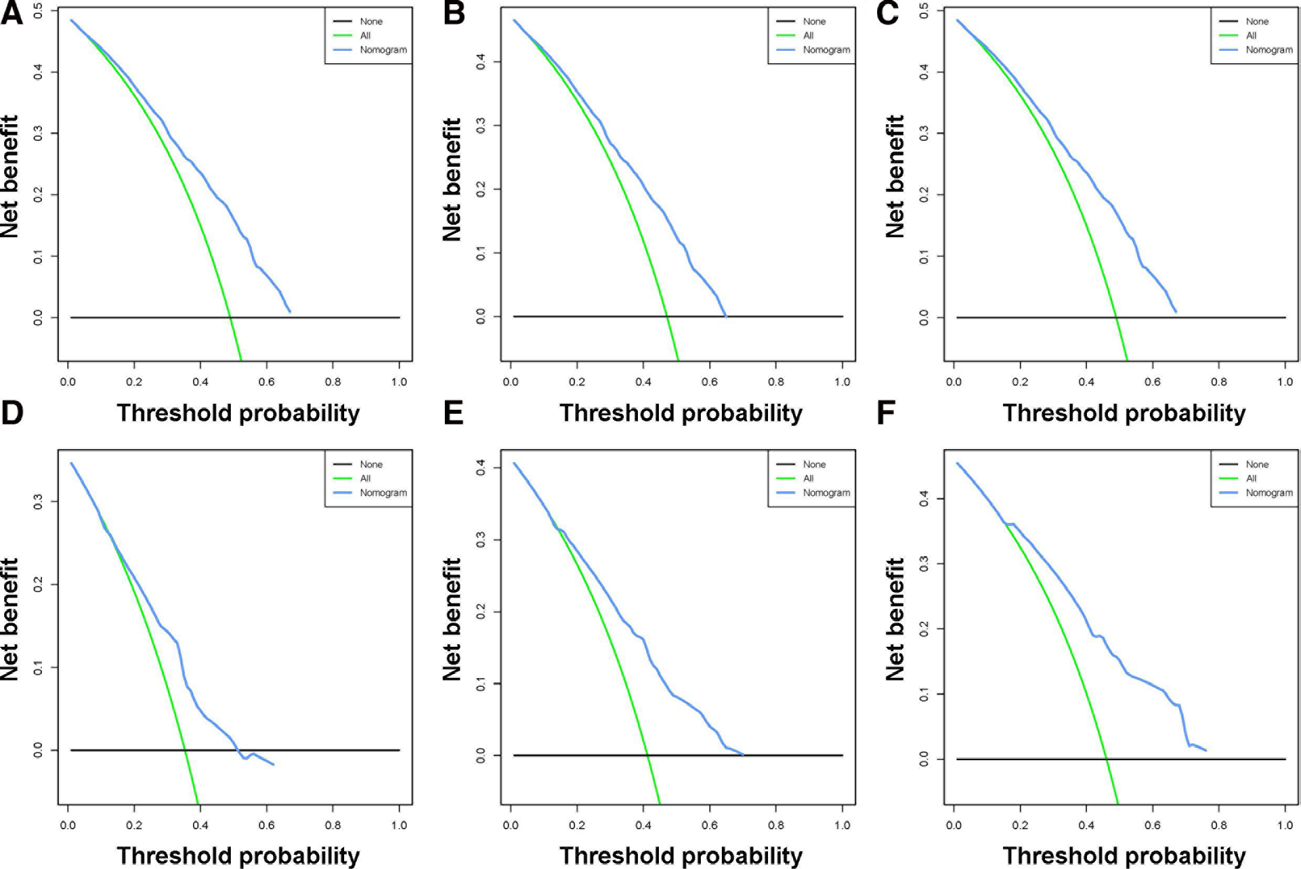
Decision curve analysis (DCA) of the constructed nomogram. (A–C) the DCA curves of 3-, 5-, and 10-yr EFS of NB patients in the training set; and (D–F) the DCA curves of 3-, 5-, and 10-yr EFS of NB patients in the validation set. DCA = decision curve analysis. EFS = event-free survival, NB = neuroblastoma.

A prognostic nomogram was created based on the 3 independent predictive factors, allowing a quantitative approach to predict the EFS of NB patients (Fig. 3). The corresponding score for each independent factor in the nomogram was obtained by drawing a vertical line to the first row (Supplementary Table 1, http://links.lww.com/MD/J632). It depicted that patient with age >1425 days, INSS stage 4, and DNA diploidy had a relatively poor prognosis. The calibration curves demonstrated a satisfactory correlation between the actual 3-, 5-, and 10-year EFS rates in the NB patients and the predicted EFS rates obtained from the constructed nomogram (Fig. 4). The area under the curve (AUCs) for the 3-, 5-, and 10-year EFS in the training cohort were 0.681, 0.706, and 0.720, respectively. Consistently, the AUCs for the 3-, 5-, and 10-year EFS in the validation cohort were 0.653, 0.703, and 0.727, respectively (Fig. 5). In addition, a comparison of the predictive accuracy between each individual independent predictive factor and the constructed nomogram was carried out (Fig. 6). As displayed in Figure 6, the AUCs of each independent predictive factor of the 3-, 5-, and 10-year EFS were smaller than those of the constructed nomogram in both the training and the validation cohorts, suggesting that the nomogram had better precision for predicting EFS in patients with NB. In addition, the DCA curves proved that the nomogram had an excellent expectation of clinical application and could serve as a simple and effective tool, helping clinical workers make better decisions (Fig. 7).

### 3.4. New event-occurrence risk stratification system

**Figure 8.**
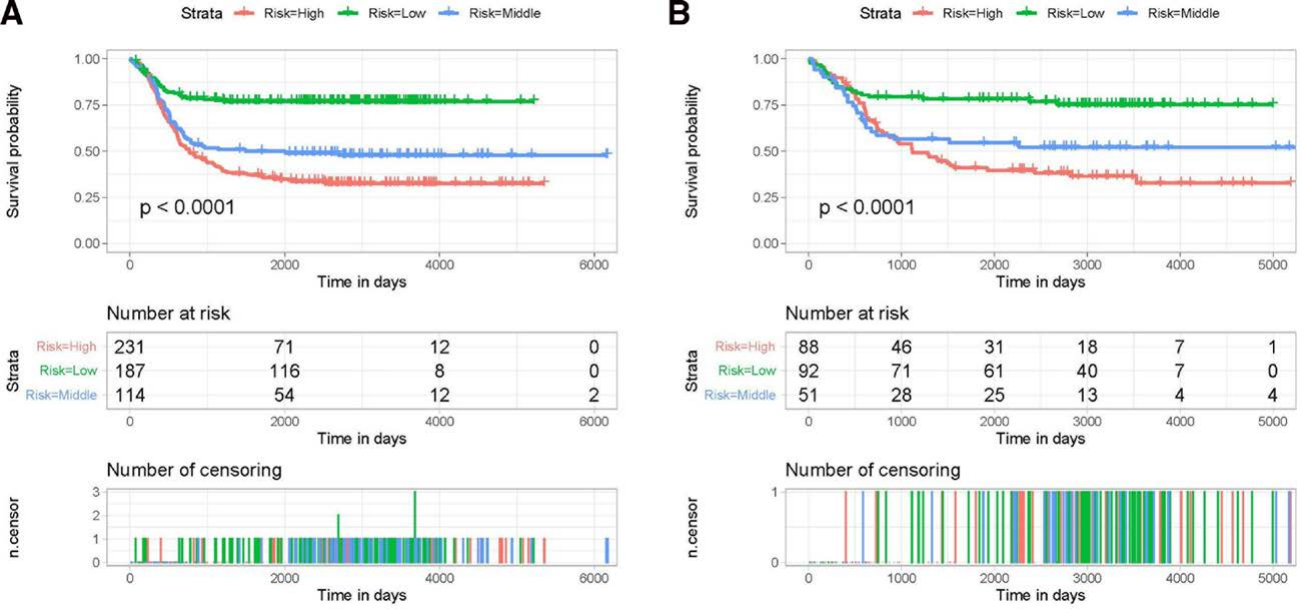
Kaplan–Meier event-free survival analysis of NB patients with different risk groups stratified by the nomogram. (A) patients in the training set and (B) patients in the validation set. NB = neuroblastoma.

To deliver individualized treatment to each NB patient, it is necessary to establish a new risk stratification system. A risk stratification system for the EFS of NB patients was developed based on the nomogram, and the overall score of the nomogram was the stratification criterion. Patients with NB were classified into 3 subgroups according to the different event-occurrence rates as follows: high- (>133), middle- (125–133), and low-risk (<125) groups based on the total score, and the most suitable cutoff value of the overall score was calculated using the X-tile software (Supplementary Figure 2, http://links.lww.com/MD/J631). In addition, the KM curves illustrated that this new stratification system could effectively classify NB patients into 3 subgroups with significant differences in both the training and the validation cohorts (Fig. 8), indicating that the nomogram could effectively differentiate the prognosis of the different subgroups of NB patients, thus improving individualized patient management.

## 4. Discussion

As the most common extracranial solid tumor in children, there are many studies related to the prognosis, diagnosis, and treatment of NB. Although there have been many studies on the prognosis of NB, most of them only involved an individual prognostic factor or focused on investigating the prognostic factors influencing the OS of NB patients rather than the CCS and EFS of NB.^[17–20]^ Most children with NB were already in the INSS stage 4 and COG high-risk group at the time of diagnosis, so their prognosis was generally poor, and they were prone to tumor recurrence or metastasis or even death after systematic treatment with surgery combined with chemotherapy.^[21]^ The recurrence and metastasis of tumors, even if they did not cause immediate death, could bring considerable trauma and burden to the physical and mental health of the children and their families.^[22,23]^ Establishing a model that can rapidly and conveniently estimate EFS for each NB patient is, in our opinion, absolutely essential.

Nomogram is a multiple-indicator combination model that predicts disease occurrence or progression in tumor survival prediction. The advantage of nomograms is that the total score is calculated based on the values of the patient predictive variables, simplifying a complex statistical prediction model involving a considerable number of variables into a simple numerical prediction model to predict the occurrence risk of an event or the probability of survival.^[24]^ In a pre-study of ours involving the NB patient data from the TARGET database, we found that 341 of the 448 (76.1%) dead patients experienced critical events such as tumor recurrence or progression before death, while 120 of the 667 (18.0%) currently alive patients had experienced such events. Therefore, to identify separate predictive factors associated with EFS outcomes, we conducted a large population-based data analysis of NB patients based on the TARGET database and developed a nomogram to predict EFS in NB patients. The constructed nomogram in this study provided a quantifiable prediction of EFS for each NB patient because it could easily incorporate the key prognostic predictors and balance the effects among them. Moreover, a new risk stratification system based on the nomogram was constructed to allow clinicians to make better choices about patient treatment.

Therefore, we investigated the influencing factors of EFS in NB patients by performing a retrospective study involving 763 patients from the TARGET database and concluded that age at diagnosis >1425 days, INSS stage 4, and DNA diploidy were independent predictive risk factors. Then, a nomogram was developed to predict the 3-, 5-, and 10-year EFS of NB. There was no significant deviation between the EFS rates of the training and the validation sets, suggesting that the nomogram has good discriminatory capability and predictive accuracy. A risk stratification system for the EFS of NB patients based on the abovementioned 3 risk factors was constructed subsequently. As determined by the nomogram overall point, NB patients were categorized into low- (<125), middle- (125–133), and high- (>133) risk subgroups, and the EFS of the 3 subgroups differed significantly (*P* < .001).

Age at diagnosis is considered an important factor affecting the prognosis of patients with various tumors, and NB is no exception.^[25]^ As early as 2005, London et al found that NB patients with a diagnosis age <18 months had a greater chance of experiencing spontaneous tumor regression and were more likely to be cured by surgery alone.^[26]^ In addition, age at diagnosis is used as an important basis for the INGRSS and COG risk subgroups. Older children with NB usually have tumors that tend to earlier recurrence; correspondingly, the prognosis of these patients is poor, which might be related to the fact that older children are more susceptible to invasive tumors that are insensitive to multimodal and cytotoxic therapy.^[25,27,28]^ In this study, age also served as one of the independent prognostic factors, and patients aged >1425 days had a significantly worse prognosis. As for gender and race, both uni- and multivariate Cox analyses demonstrated that neither of them were independent predictive factors for the EFS of NB patients (*P* > .05).

According to previous studies, tumor size and stage may affect OS in patients with NB.^[29]^ Wang et al reported that the primary tumor size was considered a key prognostic factor for NB, with tumors >4 cm suggesting a poor prognosis.^[30]^ Previously, it has been reported that NB patients with distant metastases and INSS stage 4 had considerably worse OS than patients with lower INSS stage and regional tumors.^[31,32]^ Our findings supported previous reports that higher INSS stage and COG risk groups were associated with poorer outcomes of EFS in NB patients. Both the uni- and multivariate Cox analysis identified INSS stage as an important predictor for the EFS of NB patients. These trends further demonstrated the importance of the early diagnosis and treatment of NB to improve the patient survival and reduce the rate of tumor recurrence and metastasis.

MYCN gene amplification status and DNA ploidy also had important prognostic influences on NB patients.^[33,34]^ According to previous reports, MYCN gene amplification is associated with primary giant abdominal tumors, chromosomal aberrations, and poor prognostic histological type.^[35]^ Moreno et al reported that MYCN gene status is an independent risk factors for the OS of patients with high-risk NB.^[36]^ It has also been reported that patients with DNA hyperdiploid had a better prognosis and were characterized by chromosomal instability and that the aggressiveness of tumor cells in NB might be related to the degree of chromosomal instability.^[37]^ The univariate Cox analysis in this study depicted that MYCN status and DNA ploidy were associated with the EFS of NB patients. However, the multivariate Cox analysis excluded MYCN status and considered DNA ploidy to be an independent prognostic factor.

In conclusion, the nomogram and the risk stratification system established could better predict and classify the prognosis of NB patients. However, there were some shortcomings of this study: as a retrospective study, the selection bias was inevitable; the TARGET database does not contain certain detailed diagnostic and treatment data, such as whether chemotherapy, radiotherapy, and immunotherapy were administered or other specific treatment information of patients; and the predictive accuracy of our nomogram has not been validated with patient data from other centers or databases yet.

## 5. Conclusion

NB patients with age at diagnosis >1425 days, INSS stage 4, and DNA diploid had a poor prognosis. A nomogram and event-occurrence risk stratification system were developed to predict 3-, 5-, and 10-year EFS in NB patients more easily and accurately, helping clinicians better classify different NB patients and maximize patient benefits.

## Acknowledgments

We are thankful for the contribution of the TATGET program and all the registries supplying cancer research information and all colleagues involved in the study were appreciated for their contributions.

## Author contributions

**Conceptualization:** Mingzhen Li.

**Data curation:** Xiaoying Duan, Chunyan Li.

**Formal analysis:** Xiaoying Duan, Chunyan Li.

**Supervision:** You Di, Linlin Liu.

**Writing – original draft:** Mingzhen Li.

**Writing – review & editing:** You Di, Linlin Liu.

## Supplementary Material






