# Supplementary figures and images for: A novel clinical tool and risk stratification system for predicting the event-free survival of neuroblastoma patients: A TARGET-based study
Source: Medicine (Baltimore). 2023 Sep 22;102(38):e34925. doi: 10.1097/MD.0000000000034925 (PMC10519501; doi:10.1097/MD.0000000000034925)

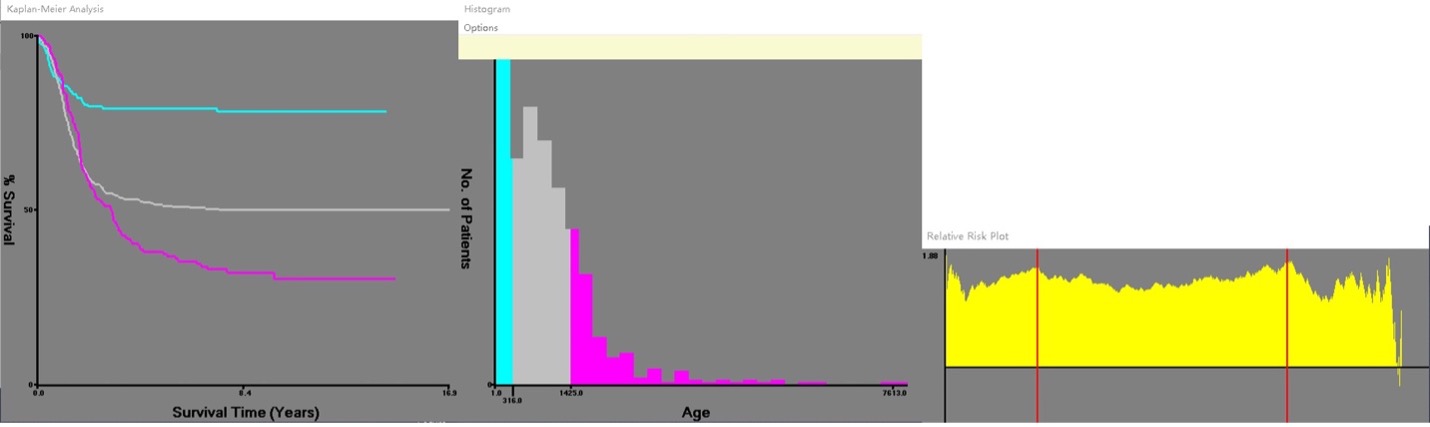

Supplement: Supplementary file 1 [file medi-102-e34925-s001.jpg]

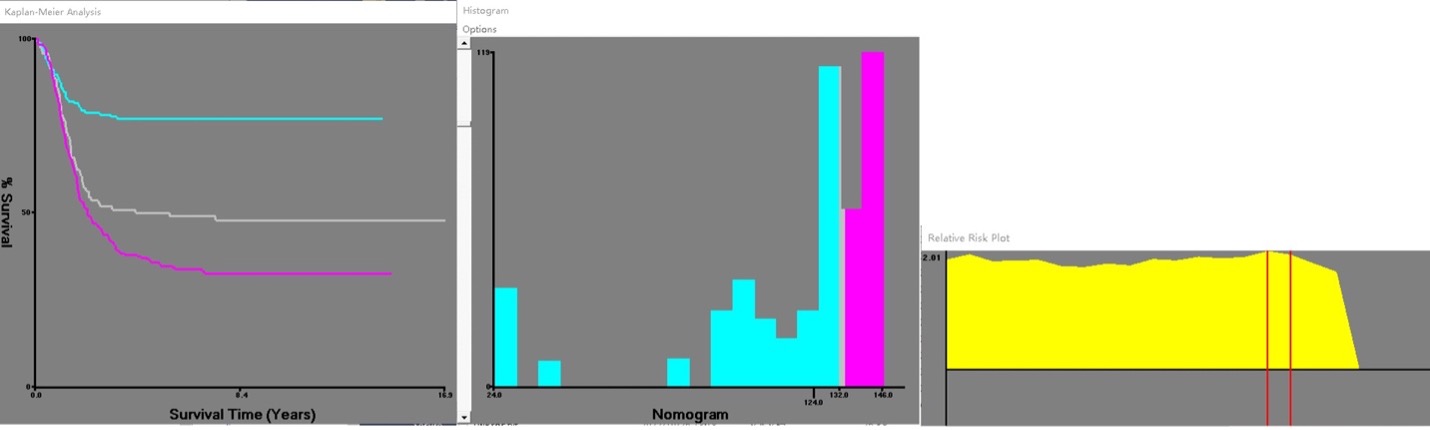

Supplement: Supplementary file 2 [file medi-102-e34925-s002.jpg]
