# Supplementary material for: A novel clinical tool and risk stratification system for predicting the event-free survival of neuroblastoma patients: A TARGET-based study
Source: Medicine (Baltimore). 2023 Sep 22;102(38):e34925. doi: 10.1097/MD.0000000000034925 (PMC10519501; doi:10.1097/MD.0000000000034925)
Supplement: Supplementary file 3 [file medi-102-e34925-s003.docx]

**Supplementary table**

**Table 1.** The point on the nomogram for each prognostic marker

| **EFS-related independent variables** | **Corresponding points in EFS nomogram** |
| --- | --- |
| **INSS Stage** |  |
| 1 | 12 |
| 2 | 90 |
| 3 | 72 |
| 4 | 100 |
| 4s | 86 |
| **Age (Days)** |  |
| <318 | 12 |
| 318-1425 | 32 |
| >1425 | 34 |
| **Ploidy** |  |
| Diploid | 12 |
| Hyperdiploid | 0 |

EFS, event-free survival; INSS, International Neuroblastoma Staging System
